# Hantzsch reaction using copper nitrate hydroxide-containing mesoporous silica nanoparticle with C_3_N_4_ framework as a novel powerful and reusable catalyst

**DOI:** 10.1038/s41598-023-36059-7

**Published:** 2023-06-12

**Authors:** Ensiyeh Rahmati, Zahra Rafiee

**Affiliations:** Department of Chemistry, Yasouj University, Yasouj, 75918-74831 Islamic Republic of Iran

**Keywords:** Chemistry, Catalysis

## Abstract

Copper nitrate hydroxide (CNH)-containing mesoporous silica nanoparticle (MSN) with g-C_3_N_4_ framework (MSN/C_3_N_4_/CNH) was fabricated via a four-step hydrothermal synthesis method. Functionalized MSN-based C_3_N_4_ was prepared, decorated with CNH, and identified by different physicochemical techniques such as FT-IR, XRD, SEM, EDX, and STA analyses. Then, MSN/C_3_N_4_/CNH composite was utilized as a robust catalyst for the fast fabrication of biologically active polyhydroquinoline derivatives with high yields between 88 and 97% via Hantzsch reaction under mild reaction conditions and short reaction time (within 15 min) owing to synergistic influence of Lewis acid and base sites. Moreover, MSN/C_3_N_4_/CNH can be straightforwardly recovered and used up to six reaction cycles without a conspicuous decrease in efficiency.

## Introduction

Multicomponent reactions (MCRs) are defined as attractive and powerful synthetic protocols for producing highly complex molecules and biological significance molecules owing to the formation of C–C and C–heteroatom bonds in a one-pot manner through an easy tandem synthetic method with step-efficiency and atom-economy^1–10^. Polyhydroquinoline (PHQ) derivatives as a significant class of nitrogen heterocycle compounds can be converted into biological compounds, displaying promising pharmaceutical and biological properties, including antitumor, antidiabetic, platelet anti-aggregation, bronchodilator, antibacterial, and neurotropic^11–14^. Thus, the production of PHQ derivatives is of great importance. The new techniques have been developed to improve the reaction efficiency in the preparation of PHQ derivatives in the presence of catalysts including [CholineCl][ZnCl_2_]_3_^15^, SBA-15@Glycine-Cu^16^, Fe_3_O_4_@SiO_2_/ZnCl_2_^17^, ascorbic acid^18^, NiAlTi LDH^19^, and CNNs-Bu-SO_3_H^20^. However, some of these synthetic methods suffer from the usage of toxic organic solvents, long reaction time, harsh reaction conditions, a great amount of catalyst, and low yields. Consequently, there is further improvement toward more sustainable protocol for the fabrication of PHQ derivatives. Lately, remarkable attention has been developed to design eco-friendly catalysts and synthetic procedures for the Hantzsch reaction. The environmentally benign processes comprise the use of effective, biodegradable, and economical catalysts and non-toxic systems such as solvent-free conditions, water, and supercritical fluids^21–25^.

The substantial advance in nanotechnology during the last decades has led to the development of a large variety of nanomaterials with outstanding catalysis applications. It is possible to design and construct numerous nanomaterials suitable as heterogeneous catalysts^26–37^. The support material selection possesses a key role in the overall efficiency of the catalyst because these materials impact the catalytic properties of nano-scale catalysts^38,39^. The materials for catalyst supports indicate the high surface area, capability to disperse the supported metal, and chemical stability. Amongst the various support materials, mesoporous silica materials (MSMs) are promising materials owing to their thermally and chemically stability, large surface area, easy surface functionalization, good biocompatibility, and can be produced with tunable micro/meso porosity^40,41^. MSMs are amorphous inorganic materials composed of silicon and oxygen elements in their framework with pore diameters ranging from 2 to 50 nm. The well-defined pore structure of porous silica can function as a molecular sieve at small sizes and may ultimately be utilized to control substrate access to the catalyst which is very important in improving/tuning the selectivity^42,43^. These materials have proved their versatility in separation^44^, sensor^45^, drug delivery^46^, and catalysis^47^. Carbon nanostructures are especially of attention owing to their promising properties including high specific surface area, excellent mechanical strength, high conductivity, and fascinating physicochemical features. Among these, graphitic carbon nitride (g-C_3_N_4_) as a free metals material is especially of attention, owing to its unique crystal structure, nontoxic, cost-effectiveness, high thermal and chemical stability, and resistance to acidic and basic conditions^48^. C_3_N_4_ has a stacked two-dimensional structure and can be synthesized easily from low-cost precursors such as urea, thiourea, melamine, and cyanamide via pyrolysis. Owing to its promising features, g-C_3_N_4_ and its composites are applied in a variety of photocatalytic applications^49^. So far, g-C_3_N_4_ has been utilized as a catalyst or catalyst support in various organic reactions^50–55^. However, the practical application of g-C_3_N_4_ is limited by its low surface area, insufficient light absorption, reduction potential, inappropriate rapid recombination, and large diffusion resistance of charges. The g-C_3_N_4_ can enhance the surface area, promote charge transfer and mass diffusion through nanostructure materials design.

Copper hydroxide nitrate, [Cu_2_(OH)_3_NO_3_], is a basic copper(II) salt with a layered structure, that have applications in vehicle airbags, catalyst, and ion exchangers^56–60^. [Cu_2_(OH)_3_NO_3_] exists as two structurally related dimorphs, a synthetic metastable monoclinic phase and a natural orthorhombic phase occurring in the mineral gerhardtite. The structure can be observed as layers of copper octahedra stacked with each other. The Cu octahedral form layers of stoichiometry [Cu_2_(OH)_3_]^+^, and NO_3_^−^ ions stand in between the positive layers for charge balance, which are linked to the hydroxyl groups via hydrogen bonding belonging to the copper octahedra layers.

In this study, g-C_3_N_4_/MSN was fabricated and utilized as a support to load copper nitrate hydroxide (CNH) (Cu_2_(OH)_3_NO_3_) and emerged as a competent heterogeneous nanocatalyst for the Hantzsch reaction.

## Experimental

### Preparation of MSN

0.2 g of glucose was dissolved in 90 mL of ethanol. Then, 4 mL of TEOS (as the silica source) and 6 mL of distilled water were added to the above solution and subsequently stirred at room temperature for 12 h. The solid was separated by centrifuge and washed with distilled water and ethanol, respectively. The obtained white solid calcined at 550 °C for 6 to the production of porous silica hollow sphere.

### Synthesis of MSN/C_3_N_4_

1.0 g of MSN, 5.0 g of urea, and 3 wt% of KBr were placed in a porcelain dish and the mixture was ground completely. Subsequently, the reaction was performed at 550 °C for 2 h in a crucible for calcination.

### Synthesis of MSN/C_3_N_4_/CNH

0.25 g of MSN/C_3_N_4_ and 0.15 g of Cu(NO_3_)_2_·3H_2_O were mixed in 40 mL of ethanol and heated under reflux conditions and argon atmosphere for 24 h. The resultant precipitate was washed (ethanol) and dried at 80 °C under vacuum for 10 h.

### The Hantzsch reaction using MSN/C_3_N_4_/CNH catalyst

#### General procedure

A mixture of MSN/C_3_N_4_/CNH (15 mg), ammonium acetate (1.4 mmol), dimedone (1 mmol), ethyl acetoacetate (1 mmol), and aldehyde (1 mmol) was stirred at 50 °C, as monitored via TLC (ethyl acetate/n-hexane 50:50) for a complete reaction. Then, 10 mL of solvent (warm ethanol) was added to the mixture and MSN/C_3_N_4_/CNH was separated via filtration. The underlying solution was heated to boiling temperature and then a piece of ice was added to precipitate the desired crystalline product. The solvent was vaporized and ethanol was utilized to crystallize the resultant product. Then, the recovered MSN/C_3_N_4_/CNH was reused in six runs under similar conditions as the first run to represent the recyclability and stability of the prepared catalyst.

## Results and discussion

### Synthesis of MSN/C_3_N_4_/CNH

**Figure 1:**
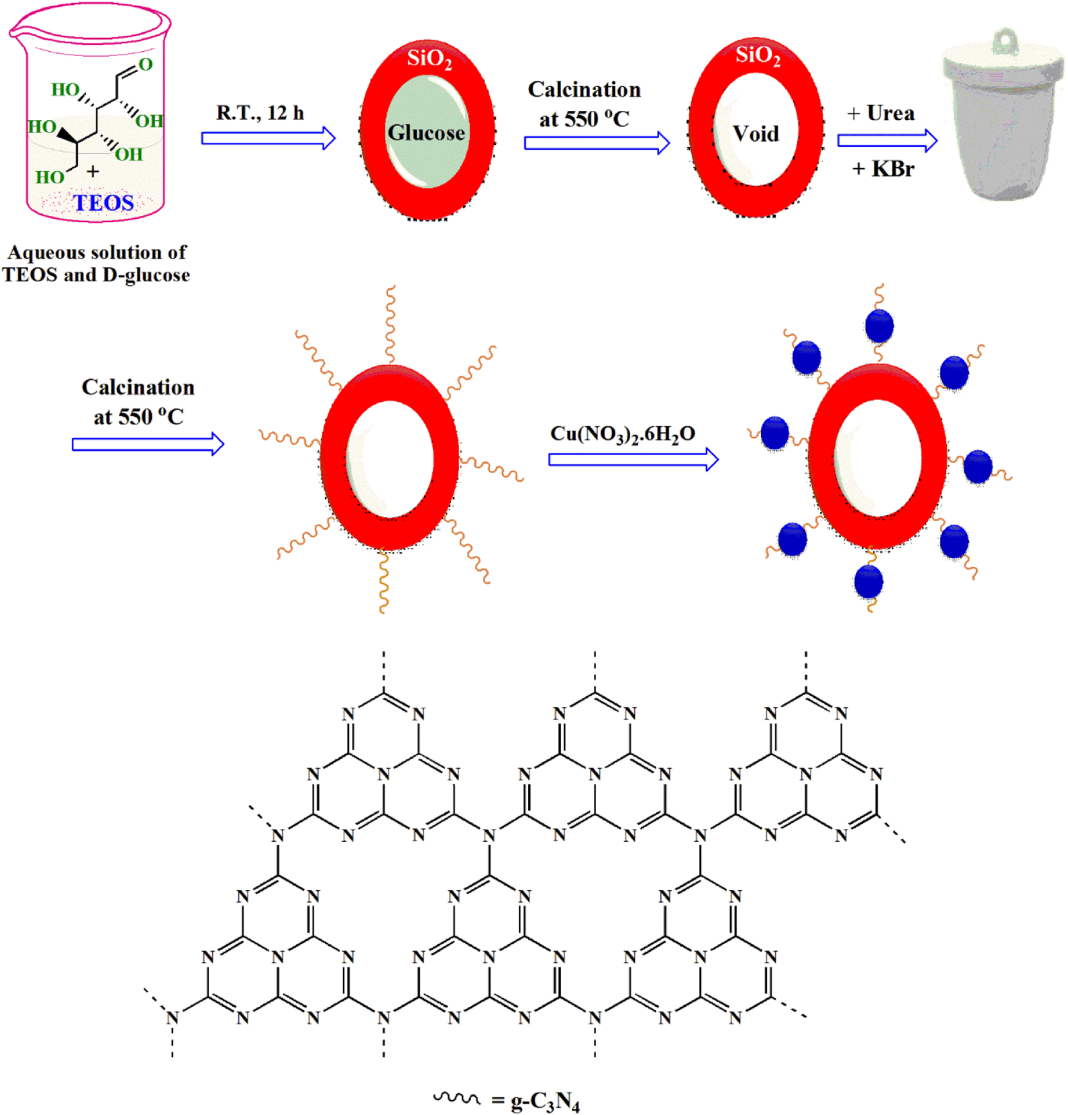
Preparation of MSN/C_3_N_4_/CNH nanocomposite.

An adequate amount of TEOS as silica precursor was added to a mixture of glucose as sacrificial template and carbon source and ethanol as a solvent. After calcination at elevated temperature, glucose was removed. MSN/C_3_N_4_ was fabricated via calcination technique onto MSN surface using urea as a precursor. MSN/C_3_N_4_ was applied as support material to anchor CNH to afford MSN/C_3_N_4_/CNH (Fig. 1).

### Characterization of synthesized compounds

**Figure 2:**
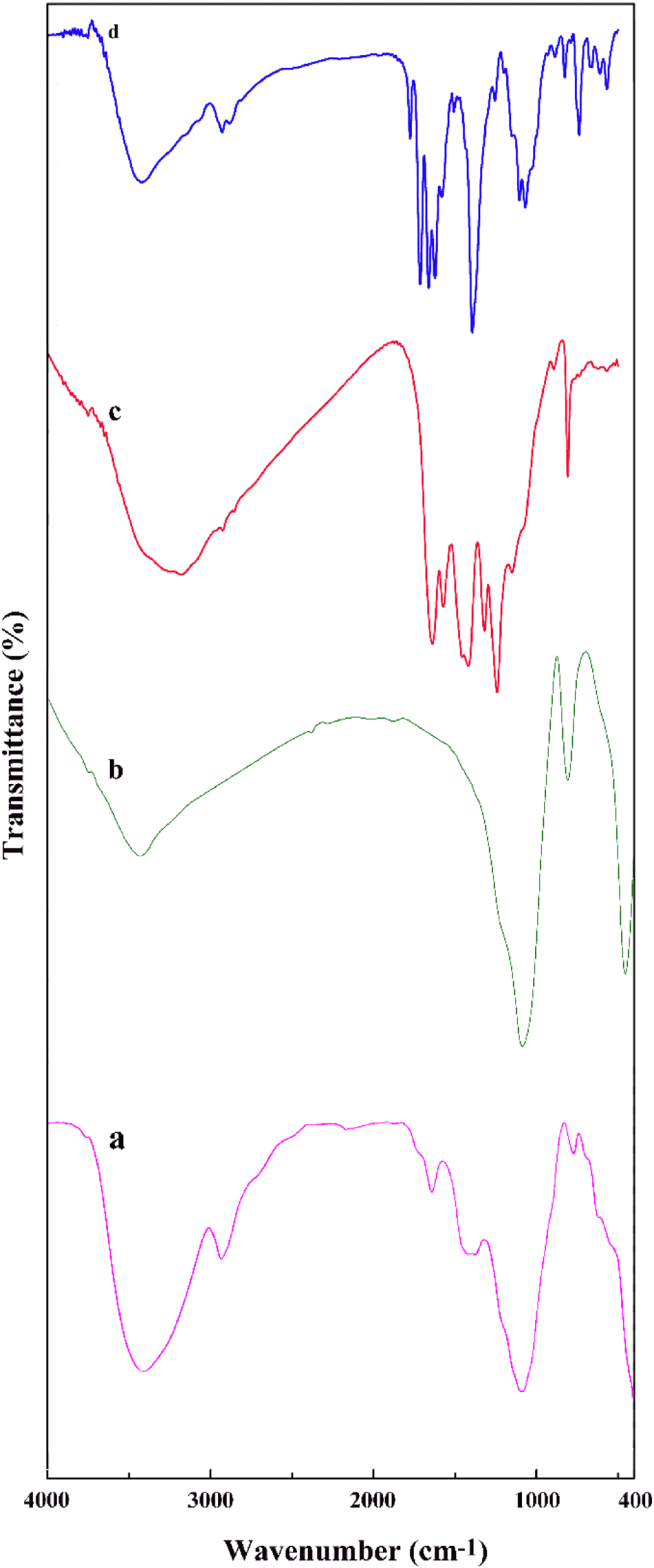
FT-IR spectra of non-calcined silica-glucose sample (**a**), MSN (**b**), MSN/C_3_N_4_ (**c**), and MSN/C_3_N_4_/CNH (**d**).

FTIR spectra of silica-glucose sample without calcination (a), MSN (b), MSN/C_3_N_4_ (c), and MSN/C_3_N_4_/CNH (d) are revealed in Fig. 2. The spectrum of non-calcined sample showed the board peak at 1087 cm^−1^ (Si–O–Si groups) and the band at 2923 cm^−1^ (C–H bonds). After calcination, the absorption peak of C–H bonds disappeared due to decomposition of templet, while the peaks of silanol and siloxane remained. In the spectrum of MSN, the peak appeared at 3429 cm^−1^ belonged to the stretching vibration of O–H; the absorption bands at 1082 and 810 cm^−1^ assigned the asymmetric and symmetric stretching vibrations of Si–O–Si, respectively. In the spectrum of MSN/C_3_N_4_, the broad band in the range of 3500–3000 cm^−1^ indicates the presence of N–H stretching vibration of the terminal amino group in g-C_3_N_4_. The peak around 1640 cm^−1^ contributed to the stretching mode of C=N bonds. The intense bands observed at 1560, 1427, 1320, and 1243 cm^−1^ were due to the presence of C–N stretching of tri-s-triazine. The band around 800 cm^−1^ reveals out-of-plane bending vibration of triazinecycle. In the spectrum of MSN/C_3_N_4_/CNH, the peak at 3427 cm^−1^ corresponds to the stretching vibration of the O–H of molecular water, and the band at 1662 cm^−1^ is owing to the bending mode of H_2_O molecules. The presence of NO_3_^−^ in MSN/C_3_N_4_/CNH is evidenced by the vibration bands that appeared from middle to lower wavenumbers, confirming the presence of mono- or polydentate nitrate ligands. The sharp absorption bands at 1052 and 1393 cm^−1^ revealing for copper nitrate hydroxide. The bands at 1384 cm^−1^ (strong) and 872 cm^−1^ are related to NO_3_ groups. The absorption band at 1052 cm^−1^ was assigned to the bending vibration of Cu–O–H. Besides, the peaks in the range of 700–500 cm^−1^ were attributed to the presence of metal–oxygen bonds.

**Figure 3:**
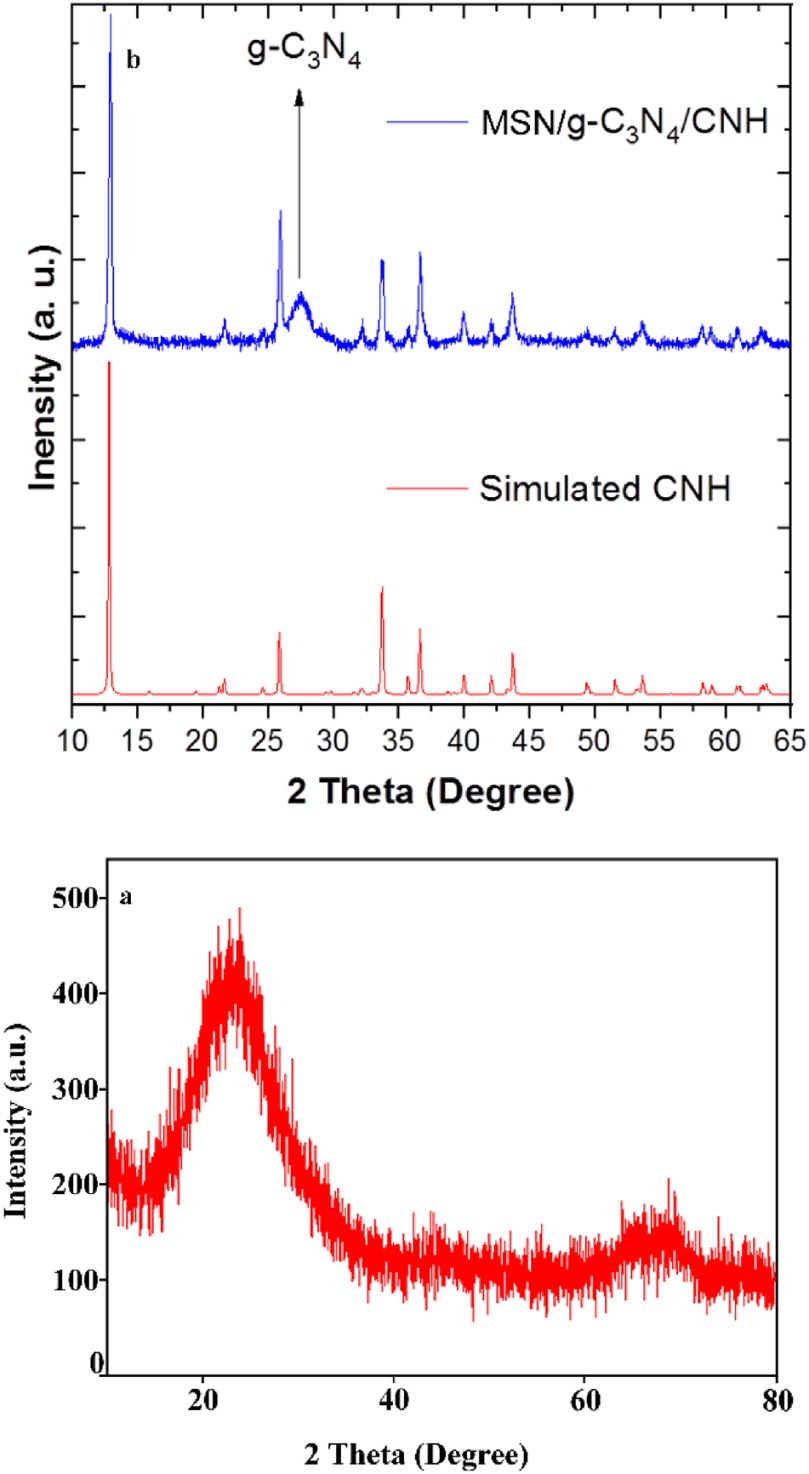
XRD patterns of MSN (**a**) and simulated CNH, and MSN/C_3_N_4_/CNH (**b**).

The XRD pattern of MSN (a) and simulated CNH, and MSN/C_3_N_4_/CNH (b) was described in Fig. 3. The XRD pattern of MSN exhibits a broad diffraction peak at approximately 22° which is characteristic of amorphous silica. In the XRD pattern of MSN/C_3_N_4_/CNH, all diffraction peaks can be well indexed to a pure phase of CNH with a monoclinic structure (JCPDS No. 74-1749). The intensive and clear peaks confirmed that MSN/C_3_N_4_/CNH nanocomposite is well crystallized. No peaks could be appeared for the impurities including Cu, CuO, Cu_2_O, Cu(OH)_2_, or Cu(NO_3_)_2_, demonstrating the high purity of MSN/C_3_N_4_/CNH nanocomposite. Furthermore, the peak at 27.5°, which corresponded to the (002) plane, was designated graphitic interlayer stacking structure of g-C_3_N_4_.

**Figure 4:**
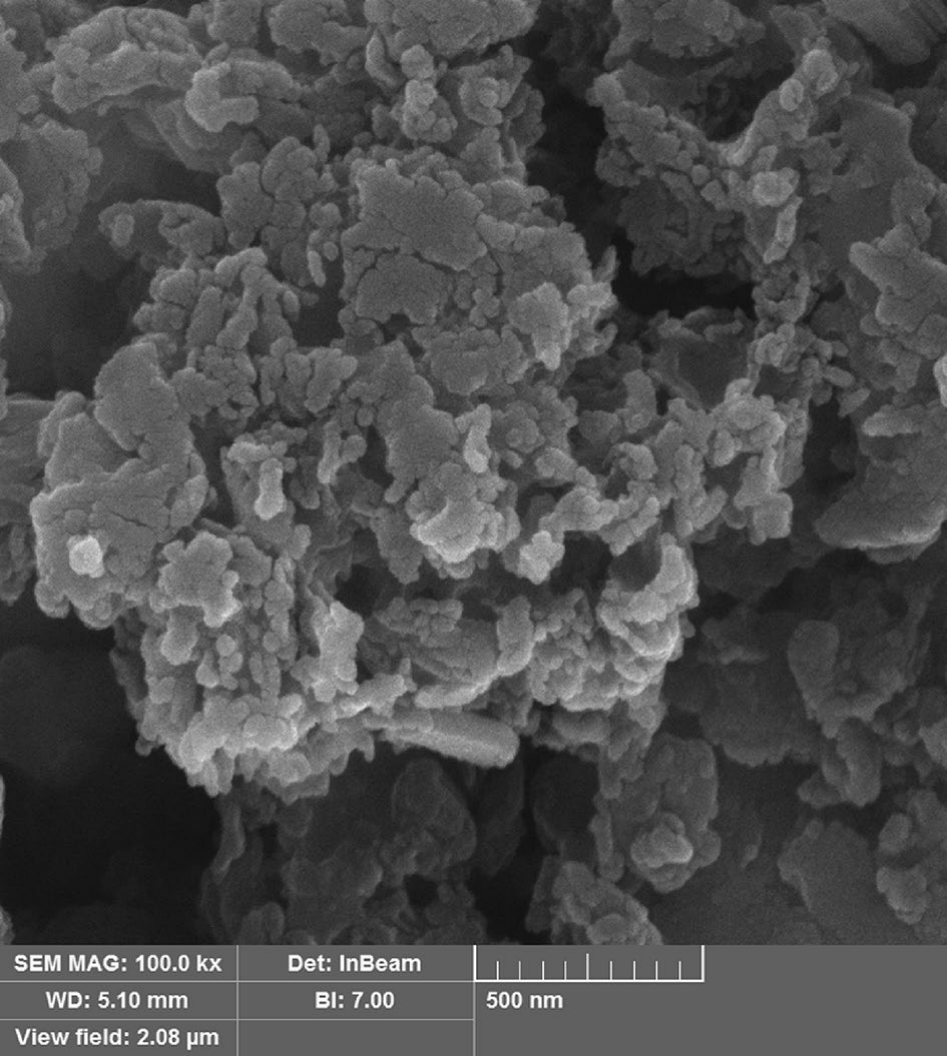
SEM image of MSN/C_3_N_4_/CNH.

In FE-SEM image of MSN/C_3_N_4_/CNH composite, spherical nanoparticles were visible, distributed uniformly over the support material with some agglomeration (Fig. 4).

**Figure 5:**
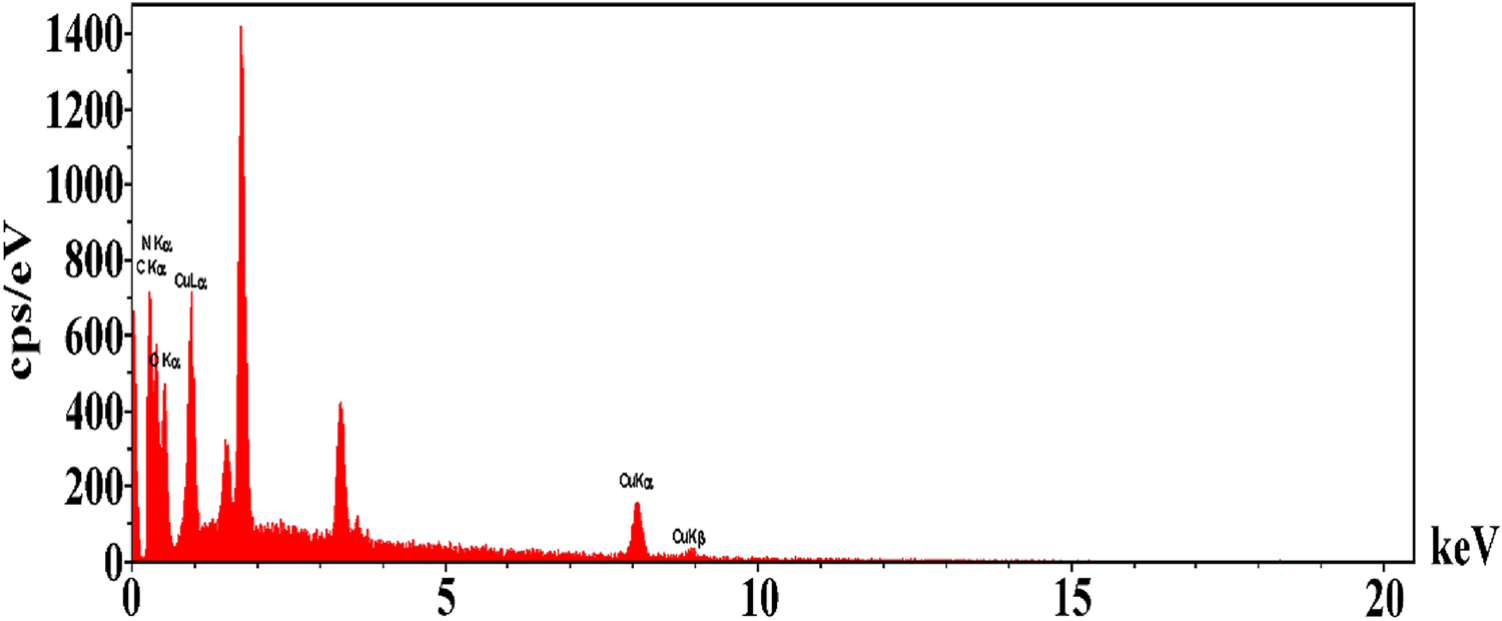
EDS of MSN/C_3_N_4_/CNH.

The average particle size was found to be around 22–38 nm. The energy dispersive X-ray (EDS) analysis proves the existence of Cu along with Si, N, C, and O elements in MSN/C_3_N_4_/CNH composite (Fig. 5).

**Figure 6:**
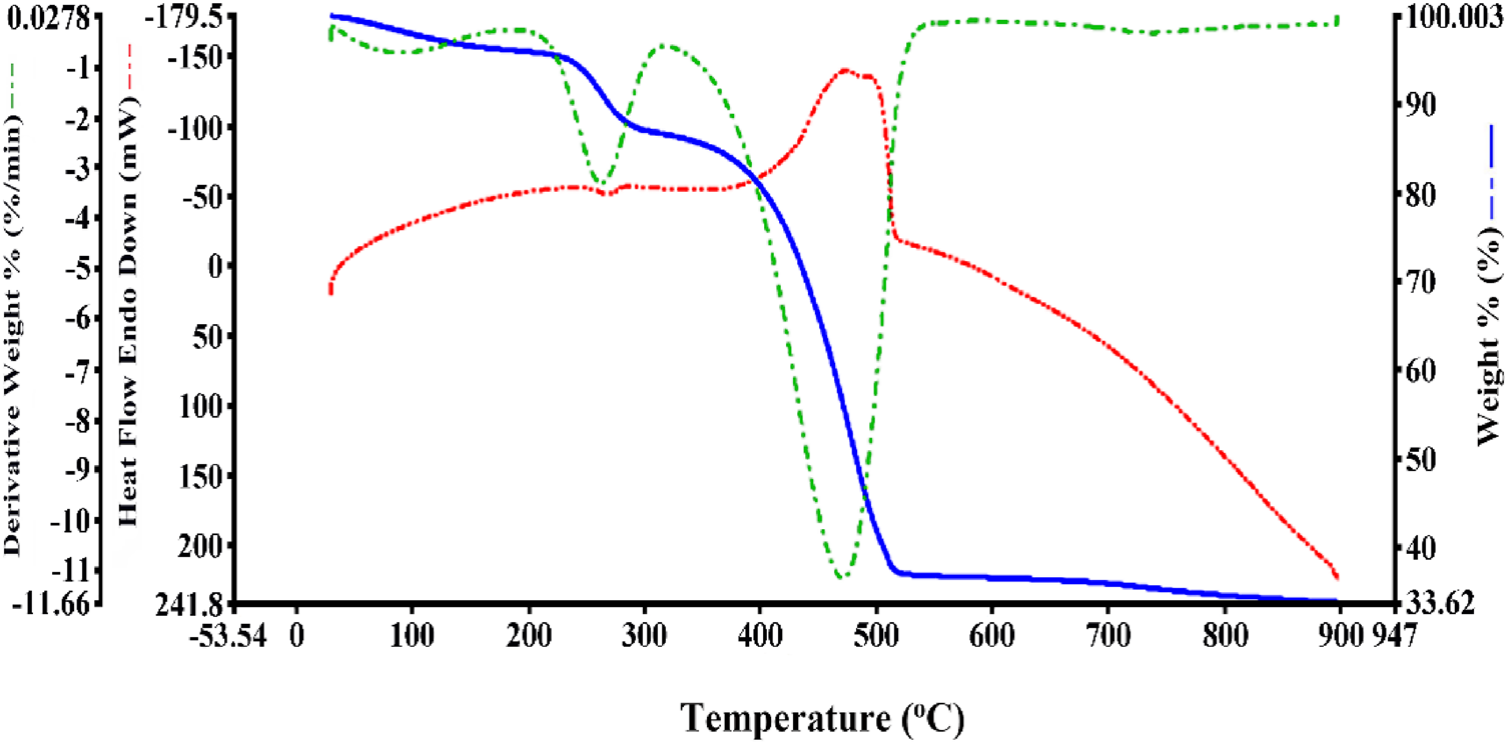
STA thermogram of MSN/C_3_N_4_/CNH.

The thermal stability of MSN/C_3_N_4_/CNH nanocomposite was examined by the simultaneous thermal analysis (STA) under a nitrogen atmosphere (Fig. 6). The initial mass loss at 125 °C is due to the evaporation of adsorbed H_2_O molecules. Between 220 and 280 °C, a mass loss is attributed to Cu_2_(OH)_3_NO_3_ decomposing into CuO and the removal of H_2_O, NO_2_, and O_2_. There is a weight loss between 390 and 520 °C, which is assigned to the combustion of g-C_3_N_4_.

### Catalytic activity test

The catalytic application of MSN/C_3_N_4_/CNH is tested in the Hantzsch reaction under diverse conditions (Table 1). The results illustrated that the reaction progress is highly affected by the amount of catalyst, temperature, and solvent. The amount of MSN/C_3_N_4_/CNH suitable to catalyze the reaction was examined by varying the amount of MSN/C_3_N_4_/CNH (5, 10, 15, and 20 mg) in the model reaction (ammonium acetate, dimedone, ethyl acetoacetate, and benzaldehyde). It was observed that the yield of the product enhanced with increasing the amount of MSN/C_3_N_4_/CNH from 5 to 10 mg (Table 1, entries 1 and 2). The best result in an appropriate time was obtained using 10 mg of catalyst (Table 1, entry 2). It is important to note that in the presence of 15 and 20 mg of MSN/C_3_N_4_/CNH the same result as 10 mg was observed (Table 1, entries 3 and 4). The efficiency of MSN/C_3_N_4_/CNH catalyst was also considerably affected by solvent (Table 1). Among the applied solvents including toluene, acetonitrile, ethanol, water, and under solvent-free conditions, the best result was obtained after 15 min under solvent-free conditions in excellent yield (Table 1, entries 5–9). Toluene delivered a low yield (25%) of the corresponding product (entry 5). Water proved to be a much better solvent in terms of yield (entry 8) than the others tested solvents including acetonitrile (entry 6), and ethanol (entry 7), which afforded the desired product in moderate yields (25–55%). With increasing temperature from room temperature to 50 °C, a dominant increase in the yield was observed (Table 1, entries 9–12). With the increasing temperature up to 70 °C, no change in product yield was observed (Table 1, entries 13 and 14).

**Table 1:** The effect of catalyst loading, temperature and solvent in the Hantzsch reaction.


Entry	Catalyst (mg)	Time (min)	T (°C)	Solvent	Yield (%)
1	5	15	50	–	47
**2**	**10**	**15**	**50**	–	**94**
3	15	15	50	–	94
4	20	15	50	–	94
5	10	15	50	Toluene	25
6	10	15	50	Acetonitrile	45
7	10	15	50	Ethanol	55
8	10	15	50	H_2_O	70
9	10	15	r.t	–	45
10	10	15	30	–	65
11	10	15	40	–	84
12	10	15	50	–	94
13	10	15	60	–	94
14	10	15	70	–	94

Significant values are in bold.

Reaction conditions: benzaldehyde (1 mmol), dimedone (1 mmol), ethyl acetoacetate (1 mmol), ammonium acetate (1.4 mmol).

The reactions of various aldehydes possessing either electron-donating or electron-withdrawing substituents with ethyl acetoacetate, dimedone, and ammonium acetate in the presence of a catalytic amount (10 mg) of MSN/C_3_N_4_/CNH afforded high yields of the corresponding polyhydroquinoline derivatives (88–97%) in a short time under the optimized model reaction conditions (Table 2). The results demonstrate that the type and position of the substituent possess no substantial influence on the activity of MSN/C_3_N_4_/CNH catalyst. The results confirm the outstanding efficiency of MSN/C_3_N_4_/CNH for the conversion of an extensive range of aldehydes.

**Table 2:** Synthesis of polyhydroquinoline derivatives by using MSN/C_3_N_4_/CNH catalyst under solvent free conditions.


Entry	R	R′	Yield (%)
1	C_6_H_5_	Et	94
2	C_6_H_5_	Me	94
3	4-NO_2_C_6_H_5_	Et	96
4	4-NO_2_C_6_H_5_	Me	95
5	4-ClC_6_H_5_	Et	96
6	2-BrC_6_H_5_	Et	97
7	4-MeC_6_H_5_	Et	90
8	4-OHC_6_H_5_	Et	92
9	3-EtO-4-OHC_6_H_5_	Et	88

Reaction conditions: aldehyde (1 mmol), dimedone (1 mmol), ethyl acetoacetate (1 mmol), ammonium acetate (1.4 mmol), catalyst (10 mg) and reaction time (15 min).

**Figure 7:**
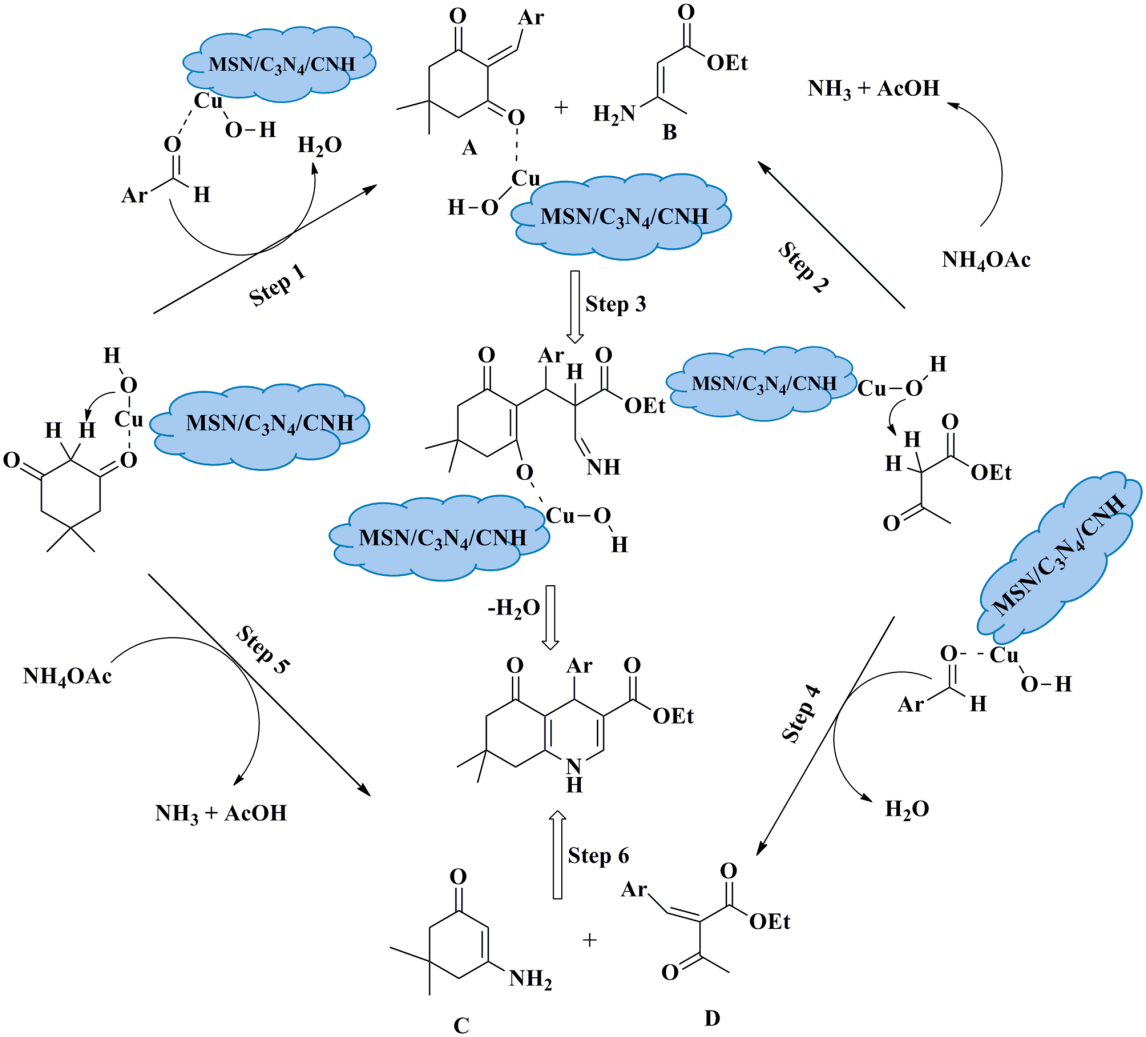
Proposed mechanism of catalytic synthesis of polyhydroquinolines using MSN/C_3_N_4_/CNH.

The proposed mechanism for the synthesis of polyhydroquinoline compounds via the Hantzsch reaction is depicted in Fig. 7. As CNH was comprised of copper hydroxide, Cu–OH bonds would exist, and Cu–OH cluster has been considered an active site for the construction of polyhydroquinoline. MSN/C_3_N_4_/CNH catalyst has both Lewis acidic sites (Cu) and basic sites (OH and C_3_N_4_), hence it is an efficient heterogeneous catalyst for the Hantzsch reaction. According to literature, Cu–OH would firstly activate the carbonyl group of aldehyde by interacting oxygen with Cu metal. The role of MSN/C_3_N_4_/CNH comes in steps 1 and 4, in which catalyzes the Knoevenagel type coupling of aldehydes with 1,3-dicarbonyl compounds and in steps 3 and 6 where it catalyzes the Michael addition of intermediates A, B and C, D to provide the corresponding product. A second important intermediate is enamine B, formed via the condensation of ammonia with ethyl acetoacetate.

### Reusability of MSN/C_3_N_4_/CNH

**Figure 8:**
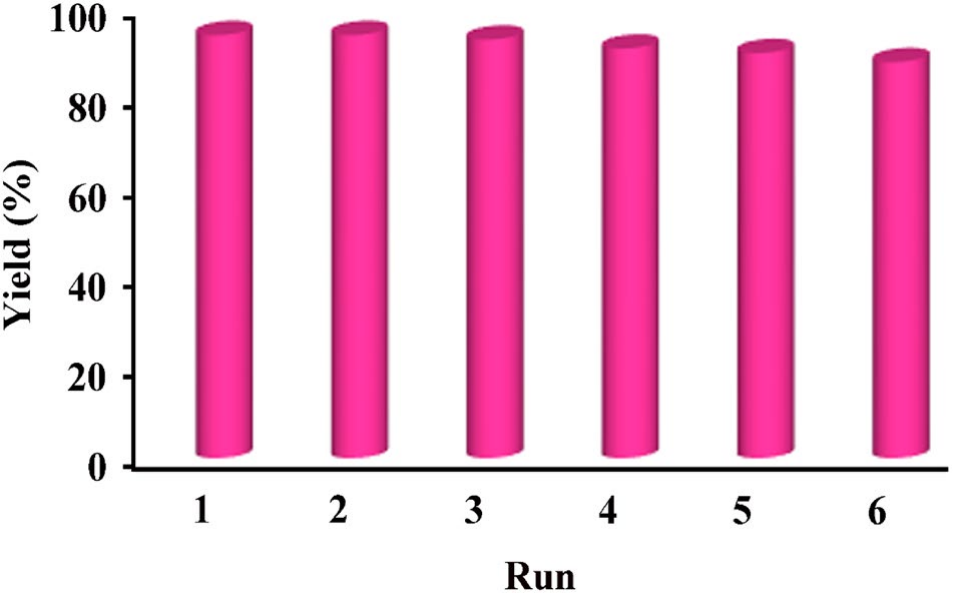
Reusability of the MSN/C_3_N_4_/CNH.

After demonstrating the activity of MSN/C_3_N_4_/CNH catalyst for the various reactions, its reusability was examined in the model reaction. In each cycle, MSN/C_3_N_4_/CNH was straightforwardly recovered, washed with ethanol, and dried at 60 °C. The reaction was repeated and the results exhibited that MSN/C_3_N_4_/CNH could be reused up to six times with a slight reduction in the catalytic activity (Fig. 8). This observation confirms the high recycling efficiency of MSN/C_3_N_4_/CNH, which is a noteworthy property from economic and environmental points of view.

### Comparison of MSN/C_3_N_4_/CNH with previously reported catalysts for the Hantzsch reaction

The performance of the MSN/C_3_N_4_/CNH catalyst was compared with that of catalysts reported in literature for the unsymmetrical Hantzsch reaction (Table 3). It is found that MSN/C_3_N_4_/CNH catalyst is superior to the majority of the reported catalysts in terms of cost-effectiveness, simplicity, short reaction time, amount of catalyst, type of solvent, and mild conditions.

**Table 3:** Comparison of the proposed catalyst with reported catalysts for the unsymmetrical Hantzsch reaction.

Catalyst	Amount	Time (min)	Solvent	Temp. (°C)	Yield (%)	Ref.
SBA-15@Glycine-Cu	80 mg	90	Ethanol	60	96	^16^
Fe_3_O_4_@SiO_2-_/ZnCl_2_	50 mg	25	–	110	90	^17^
MCM-41@PDCA-C	10 mg	10	–	100	94	^22^
IRMOF-3	4 mol%	180	–	60	91	^61^
MoO_3_ promotedCeO_2_-ZrO_2_	200 mg	45	Ethanol	reflux	93	^23^
Fe_3_O_4_@MCM-41@Cu-P2C	20 mg	200	PEG	80	92	^62^
MSN/C_3_N_4_/CNH	10 mg	15	–	50	94	Our work

## Conclusions

CNH grown on MSN/C_3_N_4_ surface was fabricated and utilized as a recoverable and powerful nanocatalyst for the one-pot construction of polyhydroquinolines in 15 min with a quantity of catalyst 10 mg at 50 °C under solvent-free conditions. The exceptional performance of MSN/C_3_N_4_/CNH catalyst can be attributed to the acid–base sites synergistic catalysis present in the catalyst. MSN/C_3_N_4_/CNH was straightforwardly recovered and reused six times with a slight reduction in the catalytic activity. The benefits of using MSN/C_3_N_4_/CNH catalyst include the low amount of catalyst, short reaction time, and solvent-free media (Supplememtary Figures).

## Supplementary Information

Supplementary Figures.

## Data Availability

The datasets used and/or analyzed during the current study available from the corresponding author on reasonable request.
