# Supplementary material for: Hantzsch reaction using copper nitrate hydroxide-containing mesoporous silica nanoparticle with C3N4 framework as a novel powerful and reusable catalyst
Source: Sci Rep. 2023 Jun 12;13:9517. doi: 10.1038/s41598-023-36059-7 (PMC10261010; doi:10.1038/s41598-023-36059-7)
Supplement: Supplementary file 1 — Supplementary Figures. [file 41598_2023_36059_MOESM1_ESM.docx]

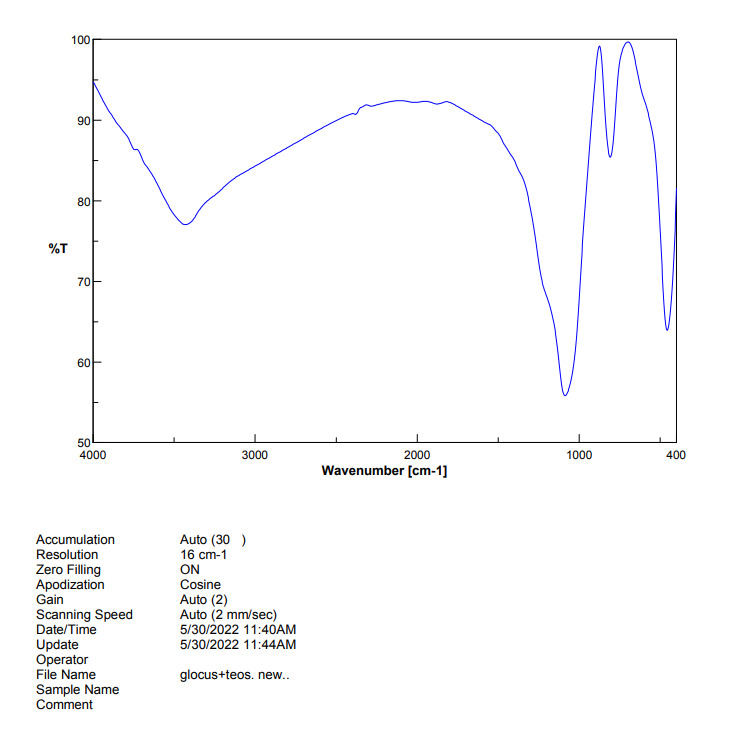


**Figure 1.** FT-IR spectrum of MSN.

**Figure 2.** FT-IR spectrum of MSN/C_3_N_4_.

**Figure 3.** FT-IR spectrum of MSN/C_3_N_4_/CNH.

**Figure 4.** XRD pattern of MSN.

**Figure 5.** XRD pattern of MSN/C_3_N_4_/CNH.


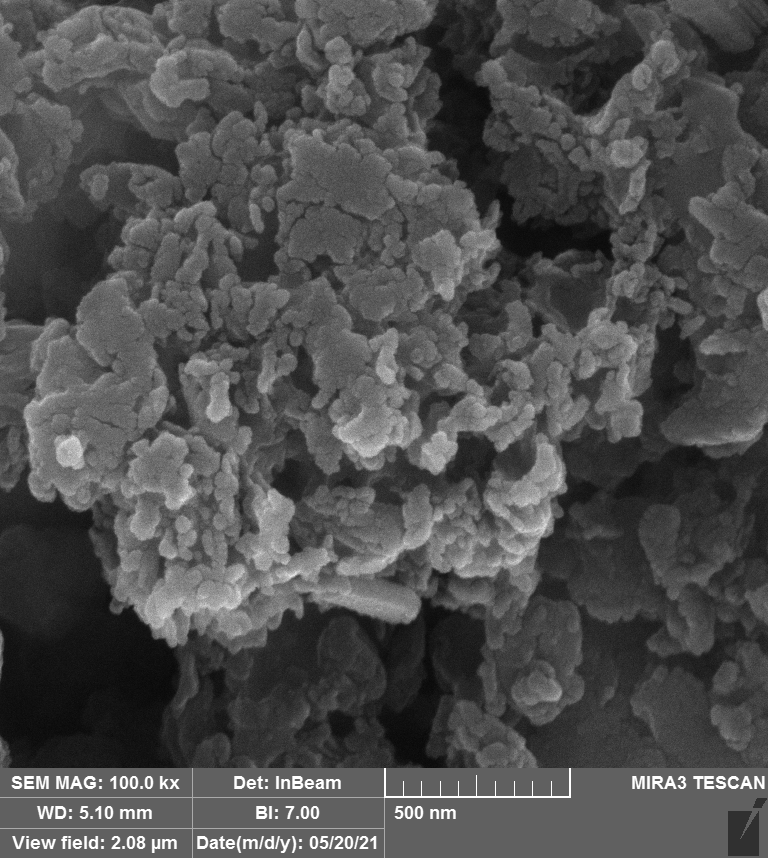


**Figure 6.** SEM image of MSN/C_3_N_4_/CNH.


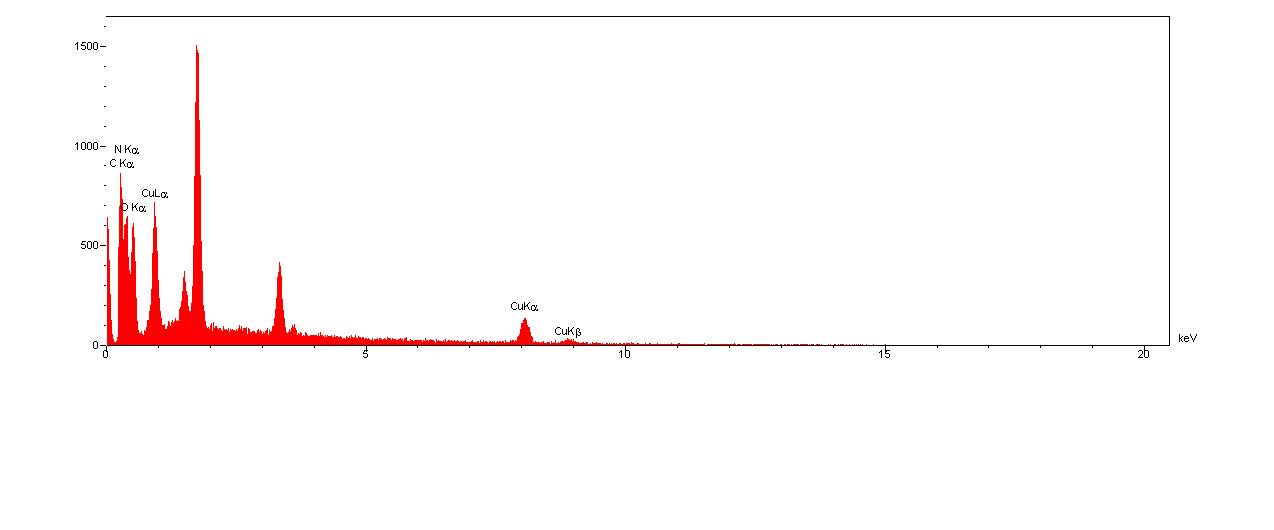
**Figure 7.** EDS of MSN/C_3_N_4_/CNH.


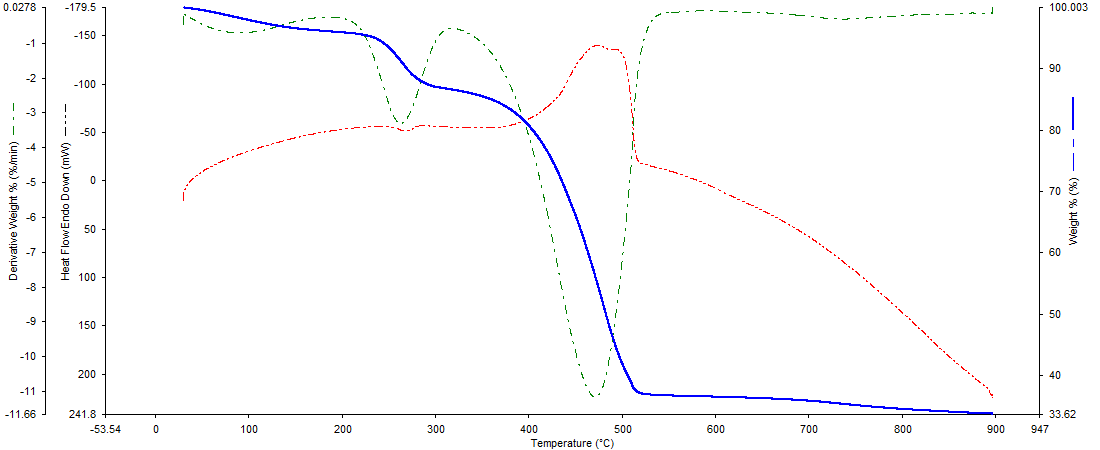


**Figure 8.** STA thermogram of MSN/C_3_N_4_/CNH.
